# Physico-chemical and bacteriological quality of drinking water of different sources, Jimma zone, Southwest Ethiopia

**DOI:** 10.1186/s13104-015-1376-5

**Published:** 2015-10-05

**Authors:** Mohammed Yasin, Tsige Ketema, Ketema Bacha

**Affiliations:** Department of Biology, College of Natural Sciences, Jimma University, P. O. Box 378, Jimma, Ethiopia

**Keywords:** Coliforms, Heavy metals, Physico-chemical parameters, MPN, Springs, Wells

## Abstract

**Background:**

The quality of drinking water has always been a major health concern, especially in developing countries, where 80 % of the disease cases are attributed to inadequate sanitation and use of polluted water. The inaccessibility of potable water to large segment of a population in the rural communities is the major health concern in most part of developing countries. This study was designed to evaluate the physico-chemical and bacteriological qualities of drinking water of different sources in the study area.

**Methods:**

The study was conducted at Serbo town and selected kebeles around the same town in Kersa district of Jimma Zone, southwest Ethiopia. Socio-demographic characteristics of the study populations were gathered using structured and pre-tested questionnaires. Standard microbiological methods were employed for determination of bacterial load and detection of coliforms. Physico-chemical analyses [including total dissolved substances (TDS), total suspended substances (TSS), biological oxygen demand (BOD), nitrate and phosphate concentrations, turbidity and electrical conductivities] were conducted following guidelines of American Public Health Association and WHO. Correlations among measured parameters of water samples collected from different water sources were computed using SPSS software (version 20).

**Result:**

Only 18.1 % (43/237) of the study population had access to tap water in the study area. More than 50 % of the community relies on open field waste disposal. Members of the family Enterobacteriaceae, *Bacillus* and *Pseudomonas* were among dominant bacterial isolates in the water samples. All water samples collected from unprotected water sources were positive for total coliforms and fecal coliforms (FC). Accordingly, FC were detected in 80 % of the total samples with counts ranging between 0.67 and 266.67 CFU/100 ml although 66.67 % of tap water samples were negative for FC. The recorded temperature and pH ranged between 20.1–29.90 °C and 5.64–8.14, respectively. The lowest and highest mean TDS were 116 and 623 mg/l, respectively. Furthermore, the mean concentration of TSS ranged between 2.07 and 403.33 mg/l. Turbidity, electric conductivity, and nitrate concentration of the water samples ranged, respectively, between 0.01–65.4 NTU, 30.6–729 μS/cm, and below detection limit to 95.80 mg/l. In addition, the mean dissolved oxygen values were found to be between 1.62 and 10.71 mg/l; whereas BOD was within the range of 8–77 mg/l. In all water samples, the concentrations of zinc were within the WHO maximum permissible limits (3 mg/l) although the lead concentration in about 66.7 % of the samples exceeded the maximum permissible limit (0.01 mg/l).

**Conclusion:**

The present study has revealed that some of the bacteriological data and physico-chemical parameters of the different water sources had values beyond the maximum tolerable limits recommended by WHO. Thus, it calls for appropriate intervention, including awareness development work and improving the existing infrastructure in order to minimize the potential health problems of those communities currently realizing of the available water sources.

**Electronic supplementary material:**

The online version of this article (doi:10.1186/s13104-015-1376-5) contains supplementary material, which is available to authorized users.

## Background

Water-borne diseases are still major health burden in many parts of the world and reported to cause about 4 billion clinical cases of diarrhea per year, representing 5.7 % of the global disease burden in the year 2000 [1]. Water is a critical component of public health, and failure to supply safe water will place a heavy burden to humanity [2]. Although poor sanitation and food are the main sources for contamination with pathogen of gastrointestinal tract, drinking water is the major source of microbial pathogens in developing regions [3]. Furthermore, water may be contaminated by disease causing pathogens from landfills and septic systems, through careless disposal of hazardous household products, agricultural chemicals, and leaking of underground storage tanks.

According to WHO estimation, about 1.1 billion people globally drink unsafe water and the vast majority (88 %) of diarrheal disease reported across the globe is attributable to unsafe water, sanitation and hygiene [1]. Furthermore, around 250 million infections each year, which results in 10–20 million deaths world-wide, occur due to water-borne diseases [4]. The wide spread of a number of diseases such as cholera, dysentery and salmonellosis are mainly due to the lack of safe drinking water and adequate sanitation that ends up in death of millions of people in developing countries every year. Diarrhea is the major cause for the death of more than 2 million people per year world-wide, majority of which are children aged less than 5 years [1].

Prior to 2004, the majority of Ethiopia’s population does not have access to safe and reliable sanitation facilities besides insufficient hygienic practices related to food, water and personal hygiene. Accordingly, more than 75 % of the health problems in Ethiopia were due to infectious diseases attributed to unsafe and inadequate water supply, and unhygienic waste management, with human excreta being the major problem [5].

Some studies conducted on bacteriological qualities of drinking water in Akaki-Kalit sub-city of Addis Ababa, Ziway, Bahir Dar and Nazareth (Adama) towns showed contamination of the water samples with indicator bacteria including total coliforms (TTC) and faecal coliforms [6]. Besides microbial contaminants, contaminations of water resources with heavy metals have received particular concern because of their strong toxicity even at lower concentration [7, 8]. Furthermore, heavy metals are not biologically degradable unlike the case of most organic pollutants, thus easily assimilated and can be bio-accumulated in the protoplasm of aquatic organisms [9]. The common heavy metals include iron, lead, arsenic, mercury, cadmium, chromium, nickel, zinc, cobalt, vanadium and copper [10, 11]. Through food chain, those heavy metals potentially reach human posing health risk to the consumer.

It could be hypothesized that untreated water could be potential sources of health risk to the local community who heavily rely on those water sources for daily consumption. The risk could be even more pronounced among unprotected water including water from wells and springs. To this effect, this study was designed to evaluate the current safety status of different water sources being used for drinking in and around Serbo town, Jimma zone, southwest Ethiopia. The water sources included in this study were tap water, protected and unprotected wells, protected and unprotected springs. Although theoretically assumed to be safe, tap water samples were collected from point of disinfection, at household levels as well as points of public services to evaluate possible challenges on the route (such as leakage or mix with sewage line) and effect of poor handling at point of services. As majority of the local community rely on alternative water sources (springs and wells), the potential health risk because of heavy dependence on these water sources (protected and unprotected) were evaluated by including both unprotected wells and springs which were accessible to both human and animal use, and those water sources protected through fencing of the water environment to lower the external interferences, were included.

## Methods

### Study site and period

**Fig. 1:**
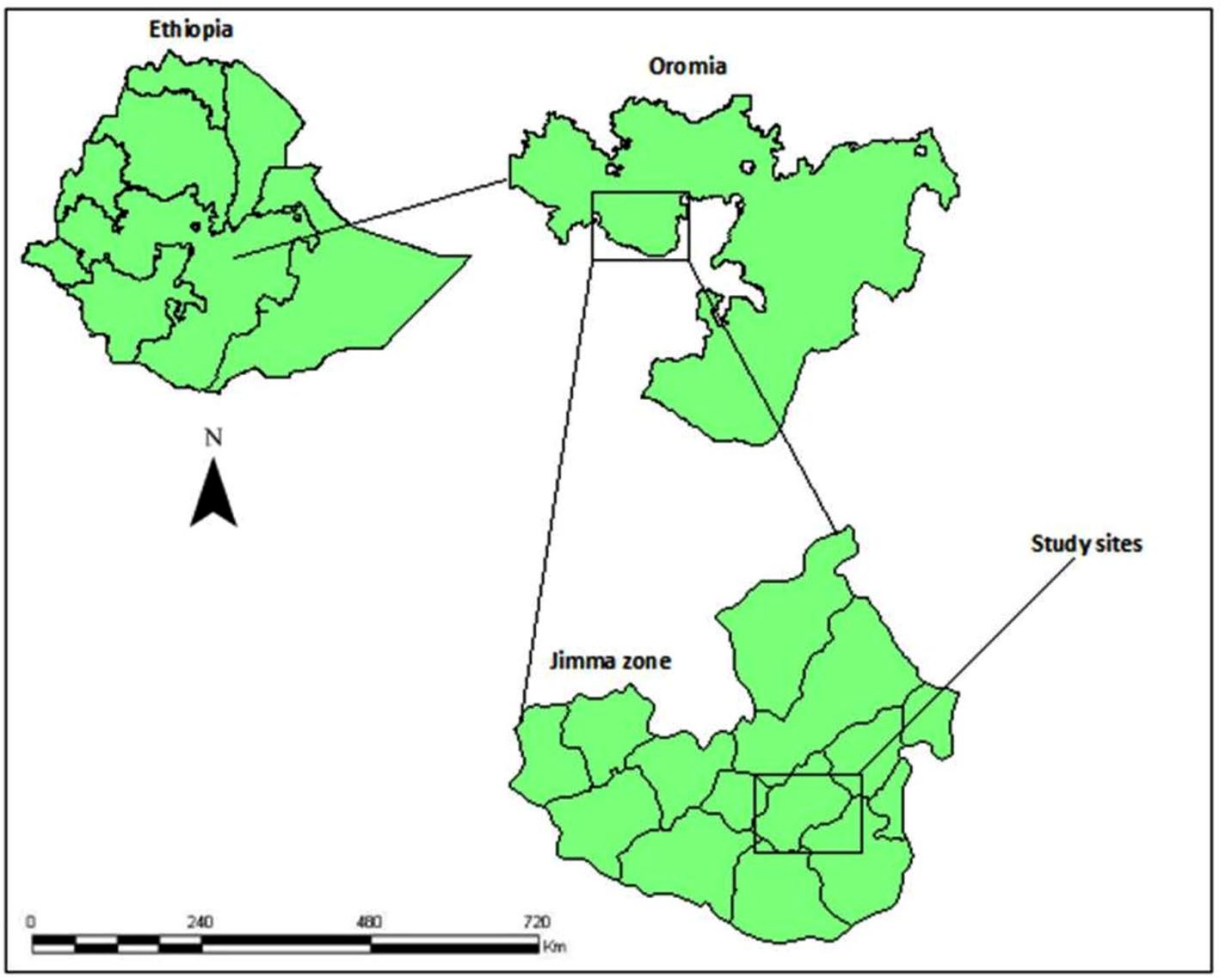
Map of the study sites, Jimma, Ethiopia, 2012

The study was conducted at Serbo town and the surrounding four kebeles (including Babo, Awaye sebu, Tikur balto and Tikur abulo) located in Kersa district, Jimma Zone, Southwest Ethiopia (Fig. 1). Serbo town is located about 332 km south of Addis Ababa, and 18 km from Jimma town, the Zonal capital. Geographically, the town is located between 7°35′–8°00′N latitudes, 36°46′–37°14′E longitude and altitude that ranges from 1740 to 2660 m above sea level. According to the 2006 census (CSA, 2006), the town has been inhabited with more than 11,855 people. The study was conducted from October, 2011 to May, 2012.

### Socio-demograpphic data collection

Structured and pre-tested questionnaires were used to gather pertinent information on socio-demographic characteristics of the study population and their level of awareness about waterborne diseases. From among 2371 households in the study area [12] a total of 237 households were included in the study, representing about 10 % of the resident population. A systematic random sampling technique was used to address representative households during socio-demographic data collection.

### Water sample collection

A total of 90 water samples were collected from five different water sources including tap water (n = 15), protected wells (n = 15), unprotected wells (n = 18), protected springs (n = 15) and unprotected springs (n = 27). Samples were aseptically collected from each sampling site in sterile glass bottles and transported to laboratory in ice box and analyzed within 6 h of sample collection. For the chlorinated water samples, about 2.5 ml sodium thiosulphate was added into each sampling bottle to stop the chlorination process during transportation.

#### Bacteriological analysis

*Isolation and enumeration* Ten ml of the water samples were separately transferred into 90 ml sterile peptone water. After thorough mixing and appropriate serial dilutions, 0.1 ml aliquot of each diluted sample was inoculated onto appropriate pre-sterilized and solidified growth medium in duplicates and spread plated on the surface of the solid agar media, incubated at appropriate temperature and time combination for the count of different microbial groups following standard procedure [13]. Accordingly, aerobic mesophilic microbes and aerobic spore formers were counted on plate count agar (PCA). MacConkey agar was used for the count of Enterobacteriaceae. For counts of coliforms and fecal coliforms, most probable number (MPN) method was employed using multiple fermentation tubes [14]. Further presumptive isolation of coliform bacteria was made on MacConkey broth. For water samples from unprotected spring, and open wells, 1, 0.1 and 0.01 ml samples were inoculated onto the first, second and third row of test tubes each containing 10 ml of single-strength MacConkey broth, respectively [15]. After incubation at 37 °C for 48 h, the tubes with acid and gas were considered positive for coliforms. From the distribution of these positive tubes, MPN of TTC was determined following standard probability table [16]. Furthermore, presence of *Escherichia coli* was confirmed by streaking loopful of broth culture onto Eosine Methylene Blue (EMB) agar and evaluating for the formation of metallic sheen color, a positive test for presence of *E. coli* [14].

*Characterization of isolates* About 10–15 colonies were randomly picked from countable plates of PCA and MacConkey agar and inoculated into 5 ml nutrient broth tubes followed by incubation at 30–35 °C for 24 h. Cultures were purified by repeated plating on nutrient agar and characterized to the genus level following standard microbiological methods. Gram reaction was determined using KOH test (test for lipopolysaccharide), the rapid method recommended by Gregerson [17]. Catalase test was performed by adding few drops of 3 % H_2_O_2_ on an overnight grown culture plate for production of air bubbles. Cytochrome oxidase test was conducted as suggested earlier [18] using freshly prepared Kovac’s reagents for detection of a blue color on freshly activated colonies within 30 s to 2 min. The appearance of blue color within the set time was considered as a positive reaction.

*Detection of Salmonella* To test for the presence of *Salmonella*, 1 ml of each sample was aseptically inoculated into 10 ml of lactose broth (LB) and incubated at 37 °C for 24 h for recovery and proliferation of cells. After the pre-enrichment, 1 ml culture was transferred into 10 ml of secondary enrichment broth (selenite cystine broth) and incubated at 42 °C for 48 h. Loopful of culture from Rappaport-Vassiliadis broth was streaked onto Salmonella–Shigella agar, Xylose Lysine Deoxycholate agar and modified Brilliant Green agar followed by incubation at 37 °C for 18 h. Characteristic colonies were picked, further purified and tested biochemically. Suspected non-lactose fermenting bacterial colonies were further characterized having inoculated into the following biochemical tubes: Triple Sugar Iron (TSI) agar, Simmon’s Citrate agar, Sulfur Indole motility (SIM) medium, Lysine Iron agar, Urea agar, and fermentation tubes of glucose, sucrose and Mannitol. Finally, the proportions of *Salmonella* positive samples were determined based on the above biochemical results.

#### Physico-chemical analysis

Turbidity was measured using Wagtech International Turbidity Meter (Wag-WT3020, Halma PLC Company), whereas other physico-chemical parameters including pH, temperature, electrical conductivity, and dissolved oxygen were measured in situ using standard instruments (HQ 40d multi parameter meter, HQ 40d, HACH Company). Biological oxygen demand (BOD), total suspended substances (TSS), total dissolved substances (TDS), and phosphate and nitrate concentrations were measured in laboratory as suggested in APHA [19]. TSS, TDS, BOD and phosphate concentration were determined according to Standard Methods 2540 D, 2540 C, 5210 B and 4500-P D, respectively, whereas Nitrate concentration was determined by phenol disulphonic acid method [19].

#### Heavy metals (lead and zinc) determination

Water samples were analyzed for presence of heavy metals (lead and zinc) using Flame Atomic Absorption Spectrometer (FAAS) [19]. Accordingly, 100 ml of the different water samples were separately digested repeatedly in nitric acid and evaporated. After the content was rinsed with de-ionized water, the resulting digest was filtered to remove some insoluble particles. The filtrate was transferred into 100 ml volumetric flask and adjusted to 100 ml with de-ionized water. Corresponding blank samples were digested in the same manner. Finally, the concentration of lead and zinc in each sample was measured using Flame Atomic Absorption Spectrometer (FAAS).

### Data analysis

Data were analyzed using SPSS statistical software (version 20). Results of physico-chemical analysis and mean microbial counts of the investigated water samples were compared with the set standards (WHO guide lines for drinking water quality) and interpreted as acceptable or unacceptable. The significances of differences within samples were determined based on calculated coefficient of variation (% CV). Mean separation between samples categories were computed using one-way ANOVA. The parameters were correlated against each other to determine their relationship using Pearson’s correlation. Variables were compared using Chi square test (χ^2^). In all cases, significance was considered at 95 % confidence interval.

## Results

### Socio-demographic characteristics of the study population

Of the total 237 respondents, the majority (32.1 %) have been using unprotected spring while equivalent proportion were relying on unprotected wells (18.6 %) and tap water (18.1 %) (Additional file 1: Table S1). Very few of them (2.5 %) were practicing boiling of water before using for drink. Plastic pots are the most favored (86.5 %) material for water storage, making the heat treatment of facilities unlikely. About 43 % of the water sources were found at a distance of less than 20 m from latrine and 32.1 % of them were located in lower elevation with respect to the nearby toilet rooms. Waste management practices of the localities was found poor as more than 50 % of the respondents dispose waste materials on open field (Additional file 1: Table S1). The Chi square test analysis revealed that, the type of water source had strong relationship with the quality of water (p < 005).

#### Microbial load of drinking water sources

The mean aerobic mesophilic count (AMC) (log CFU/ml) of tap water, protected wells, protected springs, unprotected wells and unprotected springs were 3.05, 3.53, 4.03, 4.39, and 5.25, respectively (Table 1). The highest mean Enterobacteriaceae count (4.38 ± 4.63 log CFU/ml), AMC (5.25 ± 5.84 log CFU/ml), aerobic spore formers (3.60 ± 3.49 log CFU/ml) and Fecal coliform (105.93 ± 94.92 log CFU/100 ml) were observed in unprotected springs. However, the lowest mean Enterobacteriaceae count (2.59 ± 2.65 log CFU/ml) and AMC (3.05 ± 3.12 log CFU/ml) were recorded from tap water. There were significant variations (CV > 10 %) in the count of the microbial groups within all samples and counts of both TTC and fecal coliform (FC), but the variation of TTC was not significant for unprotected spring water samples (Table 1).

**Table 1:** Mean bacterial counts (log CFU/ml or CFU/100 ml) of drinking water samples of different sources (n = 90) at Serbo town and its surroundings, 2012

Parameters		Water sample sources				
		Tap water	Protected wells	Unprotected wells	Protected springs	Unprotected springs
AMB (log CFU/ml)	Mean	3.05	3.53	4.39	4.03	5.25
	SD	3.12	3.72	4.55	4.20	5.84
	%CV	117.51	157.77	142.74	149.26	111.25
Enterobacteriaceae (log CFU/ml)	Mean	2.59	3.50	4.06	3.61	4.38
	SD	2.65	3.59	4.01	3.68	4.63
	%CV	113.96	123.25	89.29	116.13	105.69
ASF (log CFU/ml)	Mean	2.82	2.88	2.63	3.24	3.60
	SD	0.31	2.99	2.73	3.16	3.49
	%CV	11.00	128.28	125.42	84.51	97.00
TC (CFU/**100** ml)	Mean	9.67	33.00	424.89	30.60	307.06
	SD	12.93	46.95	532.90	49.56	288.25
	%CV	133.72	142.26	125.42	161.98	9.92
FC (CFU/**100** ml)	Mean	0.53	6.00	93.33	3.40	105.93
	SD	0.83	6.64	106.55	4.82	94.92
	%CV	156.34	110.73	114.16	141.84	97.65

*ASF* aerobic spore formers, *TC* total coliform, *FC* fecal coliforms, *SD* standard deviation *%CV* percent of coefficient of variation

Tap water sources had overall mean TTC and FC counts of 9.67 and 0.53 CFU/100 ml, respectively. Whereas, protected wells and protected springs had overall mean TTC counts of 33 and 30.6 CFU/100 ml, but FC counts of 6 and 3.4 CFU/100 ml, respectively (Table 1). Generally, analysis of tap water samples demonstrated that mean TTC bacterial count ranged from 2.00 ± 0.00 to 26.67 ± 19.40 CFU/100 ml, but FC ranged from 0 to 1.67 ± 0.58 CFU/100 ml. About 66.67 % of tap water samples were found to be negative for FC and *E. coli* were not detected in all the tap water samples. The entire samples from both unprotected wells and unprotected springs were positive for indicator organisms. Among the 15 protected well water samples analyzed, only 6 (40 %) had bacterial count below 10 CFU/100 ml and four (26.67 %) were negative for fecal coliforms. Sixty percent of protected springs were free from fecal coliforms and 46.67 % of these samples had TTC count less than 10 CFU/100 ml. Significant variations were observed for TTC and FC within water samples with % CV > 90 in both cases.

**Fig. 2:**
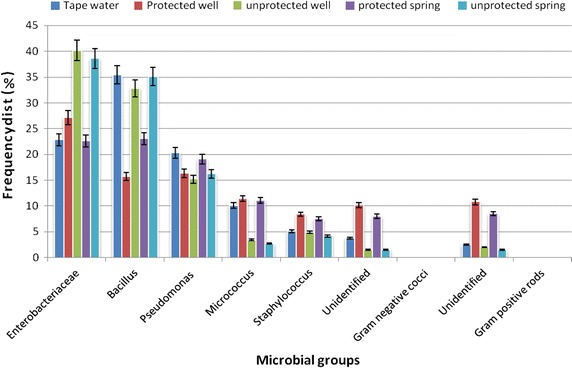
Frequency distribution (%) of dominant aerobic mesophilic bacteria in drinking water samples, Serbo town and its surroundings, 2012

A total of 907 AMB were characterized to at least group/genus levels using different biochemical tests. Accordingly, the isolates were found dominated by Enterobacteriaceae (32 %), *Bacillus* (28.4 %) and *Pseudomonas* (17 %), followed by *Micrococcus* (6.9 %) and *Staphylococcus* (6.0 %). Unidentified Gram negative cocci (4.7 %) and Gram positive rods (5 %) were among the least encountered AMB in the water samples (Fig. 2). Furthermore, from a total of 90 samples examined, only 3 (3.33 %) water samples (one from unprotected well and two from unprotected springs) were found positive for *Salmonella* spp., but all samples were negative for *Shigella* (Additional file 2: Table S2). Despite high counts of Enterobacteriaceae and coliforms in some of the water sample, the species of *Salmonella* and *Shigella* were found less prevalent.

### Physico-chemical analysis

The recorded mean temperature of the water samples were 24.42 ± 1.15, 24.53 ± 1.23, 22.79 ± 1.03, 23.52 ± 1.93 and 23.37 ± 2.16 °C for tap water, protected wells, unprotected wells, protected spring and unprotected springs, respectively (Table 2). Of the total water samples (n = 90), the maximum temperature (25.80 °C) was recorded for tap water and the minimum (20.10 °C) for unprotected springs. There were no observable significant variations both within the samples (CV = 4.52–9.24 %) and among water samples collected from the five different sources (P = 0.34).

**Table 2:** Physico-chemical analysis of drinking water samples of different sources, Serbo town and its surroundings, 2012 (n = 90)

Parameters	Water sample sources										
	Tap water		Protected well		Unprotected well	Protected spring		Unprotected spring			P- value
Temp (^0^C)		Mean	24.42	24.53		22.79	23.52		23.37	0.34	
		SD	1.15	1.23		1.03	1.93		2.16		
		%CV	4.7	5.03		4.52	8.23		9.24		
pH		Mean	7.85	6.8		6.48	6.25		6.18	0	
		SD	0.22	0.56		0.22	0.34		0.37		
		%CV	2.8	8.24		3.47	5.39		5.98		
EC		Mean	366.93	366.95		134.8	56.24		46.42	0	
(μS/cm)		SD	5.24	262.65		126.41	19.98		15.59		
		%CV	1.43	71.58		93.77	35.52		33.59		
Turbidity (NTU)		Mean	1.87	3.78		24.22	10.64		14.59	0.03	
		SD	0.43	5.78		20.45	5.68		10.06		
		%CV	22.99	152.81		84.45	53.37		68.97		
DO(mg/l)		Mean	3.96	4		3.53	5.3		5.9	0.26	
		SD	1	0.94		0.83	0.36		3.61		
		%CV	25.17	23.46		23.62	6.83		61.22		
PO_4_3^-^ (mg/l)		Mean	1.21	0.29		0.56	0.77		0.76	0.03	
		SD	0.38	0.12		0.42	0.26		0.55		
		%CV	31.27	40.06		74.63	33.64		72.02		
NO_3_- (mg/l)		Mean	1.92	42.39		8.48	5.6		2.55	0.09	
		SD	0.26	37.99		10.43	4.44		1.3		
		%CV	13.39	89.62		123.05	79.28		50.78		
TSS (mg/l)		Mean	5.93	83.01		66.33	23.47		101.08	0.25	
		SD	2.25	112.76		25.9	10.08		97.24		
		%CV	37.94	135.85		39.05	42.97		96.19		
TDS(mg/l)		Mean	524.73	415.13		348.17	327.33		137.19	0	
		SD	51.25	175.37		49.57	101.86		18.98		
		%CV	9.77	42.24		14.24	31.12		13.83		
BOD(mg/l)		Mean	9.8	34.67		62.89	22.93		35.33	0	
		SD	1.21	7.15		11.93	1.98		9.43		
		%CV	12.32	20.61		18.97	8.64		26.69		

*EC* electric conductivity, *DO* dissolved oxygen, *ND* not detected, *TSS* total suspended substances, *TDS* total dissolved substances, *BOD* biological oxygen demand

The mean pH of unprotected wells and springs were 6.48 (5.99–6.86) and 6.18 (5.64–6.75), respectively, whereas the protected wells and springs had mean pH of 6.8 (6.2–7.77) and 6.25 (5.79–6.62), respectively. Tap water samples had mean pH value around neutrality (pH = 7.85) ranging between 7.4 and 8.14. Statistically significant mean variations were observed among the water samples collected from five different sources (p < 0.05) although there was no significant differences within same sample source (CV < 10 %).

Mean electric conductivity (μS/cm) for tap water, protected wells, unprotected wells, protected spring and unprotected springs were 366.93 ± 5.24, 366.95 ± 262.65, 134.80 ± 126.41, 56.24 ± 19.98 and 46.42 ± 15.59, respectively (Table 2). There was a statistically significant difference (P < 0.05) among mean electric conductivities of different water samples and within samples (except for tap water).

The mean turbidity value of water samples was the highest (24.22 NTU) for unprotected wells and the least (1.87 NTU) for tap water. The high turbidity observed in some of the water sources did not agree with WHO standards (5 NTU). Variations were statistically significant within samples (CV > 10 %) and among means of different water samples (P = 0.03) (Table 2).

The mean valves of dissolved oxygen (DO) (mg/l) for tap water, protected wells, unprotected wells, protected spring and unprotected springs were 3.96 ± 1.00, 4.00 ± 0.94, 3.53 ± 0.83, 5.30 ± 0.36 and 5.90 ± 3.61, respectively (Table 2). There was no significant differences (P = 0.264) in DO among the assessed water samples. Similarly, mean phosphate concentration (mg/l) level recorded for tap water, protected wells, unprotected wells, protected spring and unprotected springs were 1.21 ± 0.38, 0.29 ± 0.12, 0.56 ± 0.42, 0.77 ± 0.26 and 0.76 ± 0.55, respectively (Table 2). Phosphate concentration did not show significant variations (p = 0.31) among water samples although highly variable within samples (CV > 10 %).

Mean nitrate concentration (mg/l) values of 1.92 ± 0.26, 42.39 ± 37.99, 8.48 ± 10.43, 5.60 ± 4.44 and 2.55 ± 1.30 were recorded, respectively, for tap water, protected wells, unprotected wells, protected spring and unprotected springs (Table 2). The maximum mean nitrate value of 95.80 ± 8.45 mg/l was recorded from protected well and the minimum from protected wells and protected spring with records below detection level of the instrument used (data not given). Variations were not statistically significant among means of different water samples (P = 0.09). Likewise, the mean TSS (mg/l) of tap water, protected wells, unprotected wells, protected spring and unprotected springs were 5.93 ± 2.25, 83.01 ± 112.76, 66.33 ± 25.90, 23.47 ± 10.08 and 101.08 ± 97.24, respectively (Table 2), the highest mean concentration being in unprotected spring and the least in tap water. The highest mean TSS concentration of 305.00 ± 14.14 (mg/l) was obtain from unprotected spring, whereas the lowest 2.67 ± 0.58 (mg/l) from tap water source. Statistically significant variations were not observed among mean values s of different water sampling sources (P = 0.25) but within all samples of the same source.

Unusually high TDS level (524.73 ± 51.25) was observed in tap water samples while relatively lowest level (137.19 ± 18.98) was encountered in unprotected well water. Variation in TDS within samples was not significant (% CV < 10). From the total sampling sites, 623.00 ± 10.54 mg/l was the highest total dissolved solids (TDS) recorded from one of the protected well while the lowest concentration (116.00 ± 12.00 mg/l) was recorded from unprotected spring (data not shown). Significant variations were noted among the five different water sample sources (P < 0.05) and variation within sample was not significant for tap water.

The observed BOD value (mg/l) was the highest in unprotected well (62.89 ± 11.93) followed by unprotected spring (35.33 ± 9.43), protected well (34.67 ± 7.15), protected spring (22.93 ± 1.98) and tap water (9.8 ± 1.21) (Table 2). The lowest mean BOD value was 8.33 ± 0.58 mg/l from private tap water sample, whereas the highest mean value (74.67 ± 2.52 mg/l) was recorded from unprotected well (detailed data not shown). There were statistically significant variations in BOD values among different water samples collected from the five sources (P = < 0.05).

In relation to the abundance and concentrations (mg/l) of the two heavy metals (lead and zinc) in the drinking water samples, relatively higher concentration was recorded in tap water and unprotected wells (0.03 ± 0.03 each) and almost similar concentration observed in protected wells (0.02 ± 0.01), protected spring (0.02 ± 0.01) and unprotected springs (0.02 ± 0.03) (Table 3). Maximum lead metal concentration of about 0.09 mg/l was observed in tap water. There was no statistically significant variations among the mean concentrations of the different water sampling sources (P = 0.644). Relatively higher zinc concentrations of about 0.41 and 0.27 mg/l were recorded from tap and protected well water samples, respectively, minimum values below detection level. Variations were statistically significant among means of different water sampling sources (P = 0.003).

**Table 3:** Concentration of some heavy metals in drinking water samples, Serbo town and its surroundings, October (2011)

Heavy metal	Conc.	Water source						Standards	
		Tap water	protected wells	Unprotected wells	Protected spring	Unprotected springs	p- value	WHO	USEPA
Lead (mg/l)	Mean	0.03	0.02	0.03	0.02	0.02	0.64	0.01	0.015
	SD	0.03	0.01	0.02	0.01	0.03			
	%CV	104.1	34.40	55.86	91.88	176.47			
	Min	BDL	0.01	0.00	BDL	BDL			
	max	0.09	0.04	0.07	0.04	0.08			
Zinc (mg/l)	Mean	0.19	0.13	0.02	0.02	0.03	0.003	5.00	5.00
	SD	0.13	0.11	0.02	0.03	0.04			
	%CV	67.28	87.81	108.74	174.32	141.90			
	Min	0.06	0.02	0.00	0.00	BDL			
	max	0.41	0.27	0.06	0.07	0.14			

*BDL* below detection level

#### Association between physico-chemical parameters and microbial loads

The correlation analysis indicated that AMC was positively correlated with turbidity, DO and total suspended solids (TSS) (r = 0.721, r = 0.626, and r = 0.718, respectively) (Additional file 3: Table S3); and negatively correlated with pH, EC and TDS (p < 0.05) (r = −0.829, r = −0.845 and r = −0. 813, respectively) (Additional file 3: Table S3). Temperature had negative correlation with turbidity and BOD (r = −0.987, p < 0.05 and r = −0.985, p < 0.05), respectively. The values of pH positively correlated with TDS and EC; p < 0.05, but negatively correlated with TSS. Furthermore, electric conductivity and turbidity values were positively correlated with total dissolved solids (TDS) (r = 0.831), and BOD (r = 0.860) (p < 0.05, in both cases).

## Discussion

The mean AMC of tap water (3.05 log CFU/ml) and protected well water (3.53 log CFU/ml) samples documented in this study, with about 70 % of the water samples having aerobic AMCs greater than 3 log CFU/ml, was in agreement with the earlier report from Nigeria [20]. Although the observed contamination level with regards to aerobic mesophilic bacteria was not significantly high, their very detection by itself is an indication of high vulnerability of the water sources to microbial contamination, including potential pathogens.

The predominant bacterial groups identified in the water samples were members of the family Enterobacteriaceae, *Pseudomonas* spp. and *Bacillus* spp. Similarly, other scholars [21] reported that the most prevalent bacterial species in well water sources from Rural Areas of Zimbabwe were members Gram negative, non-spore forming bacilli belonging the family Enterobacteriaceae. In agreement with the report made by earlier [22], *Bacillus* species were the second dominant bacterial groups in the current study. Few of the *Bacillus* species, including strains of *Bacillus cereus*, are pathogenic to humans and animals being responsible for food poisoning [23]. The incidence of *Pseudomonas* spp. as the third dominant bacteria in the current study was in agreement with report made elsewhere [24, 25].

With 100 and 80 % detection rates of TTC and thermo-tolerant coliforms, respectively, about 76.67 % of the samples had TTC bacterial count beyond the Canadian acceptable level for drinking water (10 CFU/100 ml) [26] with all water samples having microbial counts above WHO recommendation (0 CFU/100 ml) [27]. According to WHO guidelines, *E. coli* or thermo-tolerant coliform bacteria should not be detectable in any water intended for drinking [15, 28]. Results of this study were in agreement with the reported detection of coliforms from 75 % of unprotected well and spring samples from North-Gondar, Ethiopia [29] and the 90 % detection of the same microbial groups from protected spring samples of Uganda [30]. Similarly, 87.5 % of the water samples collected from other six protected wells and eighteen unprotected wells of Serbo town [31] revealed TTC count above the permissible limits for drinking water.

About 80 % of the water samples were positive for fecal coliforms (FC) and the highest observed mean coliform count was 266.67 CFU/100 ml. In contrary to our report, significantly high counts (1100 CFU/100 ml) of FC bacteria were reported from water samples collected from rural areas of Iran [32] and unprotected springs of central highlands of Ethiopia (741.7 CFU/100 ml) [33].

The prevalence of *Salmonella* was very low in the current study, with only two positive samples from unprotected springs and one from unprotected well water samples. In a related study, Shittu et al. [34] reported absence of *Salmonella* and *Shigella* in all well water samples examined in Nigeria although stream samples were positive. However, as long as the counts of fecal coliforms are high in most of the water samples examined for microbial load and safety, the absence of any *Salmonella* and *Shigella* in many of the samples could not qualify the water sources’ safety.

Temperature is one of the physico-chemical parameters used to evaluate quality of potable water. It affects many phenomena including the rate of chemical reactions in the water body, reduction in solubility of gases and amplifications of tastes and colours of water [35]. The highest (25.73 °C) and lowest (20.67 °C) temperature recorded from tap water and unprotected spring, respectively, were related to the 28 °C reported from different water source of Nigeria [13] but higher than the study conducted in Bahir Dar town (15–20 °C) [36]. Almost all the recorded water temperatures were above the WHO recommended level (<15 °C) and temperature optima of some aerobic mesophilic bacteria and fungi. The variations in temperature of the samples may be attributed to sampling locations as some of the water sources were collected from underground (including well water) while others were found partly on the surface exposed to direct sunlight. Richness in organic matter, hence microbial activities, could also contribute besides the geographic location of the study area (tropical zone). It is desirable to have the temperature of drinking water not exceeding 15 °C as the palatability of water is enhanced by its coolness [10].

With the overall mean pH value of 6.72 (ranged between 5.72 and 8.14), only about half (52.3 %) of the pH of water samples fall within WHO standard (6.5–8.5) [37]. Except tap water, the majority of other drinking water sources were slightly acidic (below pH of 7), whereas tap water sources had pH value greater than 7 (slightly alkaline). The pH values in most of the samples were found within the recommended standards of European Commission and WHO (ranges from 6.5 to 8.5) for potable waters. According to Byamukama et al. [38], the low pH values observed in most wells and springs could be associated with carbon dioxide saturation in the groundwater. In fact, the physico-chemical nature of the soil of sampling sites could partly contribute to the final pH of the samples. In related development, the pH of water samples collected and analyzed from Katanga, North of Kampala city, were found to be acidic [39] contributing to the final low pH of water samples analyzed from the same sites.

In this study, about 60 % of the samples had turbidity level above 5 NTU (beyond the acceptable standard) although all tap water and 80 % of the protected wells had values below 5 NTU. High turbidity is often associated with higher levels of suspended organic matter and microorganisms including bacteria and other parasites. Usually, the acceptable turbidity level is 5 NTU although it could vary with local circumstances [15]. The consumption of highly turbid water may constitute a health risk as excessive turbidity can protect pathogenic microorganisms from the effect of disinfectants, and also stimulate the growth of bacteria [40].

The highest conductivity recorded from tap and protected water sources could be due to the corrosion of metals that led to the accumulation of heavy metals. Even though conductivity values in the water samples ranged from 30.77 to 727.67 μS/cm, more than 93.33 % of the samples had electric conductivity (EC) value below 399 μS/cm, with the lowest conductivity values recorded from protected and unprotected springs. Actually all mean EC values were within WHO maximum recommended limit (1500 mg/l). Related results were reported from well water samples in Nigeria [13], where the EC levels ranged from 22 to 315 μS/cm. However, EC values greater than our finding was found in ground water sources of Turkey, where the lowest and highest conductivities were 463 and 1460 μS/cm, respectively [41].

The lowest total dissolved solid (TDS) (116 mg/l) recorded from unprotected spring and the highest value (623 mg/l) recorded from protected well were below the maximum allowable limit (1000 mg/l) recommended by WHO [37]. Total dissolved solid (TDS) are measures of the general nature of water quality [35]. The TDS include carbonate, bicarbonate, chloride, sulphate, phosphate, nitrate, calcium, magnesium, sodium, organic ions and other ions. TDS affect the taste of drinking water if present at levels above the WHO recommended level. Accordingly, the TDS values recorded in this study could be considered tolerable. On the other hands, the overall mean total soluble substances (TSS) recorded in the study ranged between 5.93 and 101.08 mg/l with the lowest and highest measurements being observed in tap water (2.67 mg/l) and the lowest in unprotected spring water samples (403.33 mg/l). The variability or range in the recoded TSS data was significantly high as compared to the earlier report (10–32.4 mg/l) made from Southern Rajasthan, India [42] from hand pump water sources and the 210.0 ± 127.7 mg/l from untreated tap water of Jimma town, Ethiopia [43]. Although there is no set guideline for the maximum permissible limit of TSS in drinking water, the TSS value recommended for fisheries and aquatic life in Ethiopia (25 mg/l) could be used as reference for this purpose [43]. Accordingly, the concentrations of TSS obtained from all unprotected wells, most of unprotected spring (85.2 %) and protected spring (80.0 %) water sources were above even the tolerable limits for maintenance of aquatic life and fisheries. The higher concentration of TSS in the water samples could be due to poor sanitation practice with possibility of contamination of the water sources with municipal wastes and plant debris.

The different water samples revealed mean dissolved oxygen (DO) values ranging between 3.53 and 5.9 mg/l although there were significant variations both within and among samples. About 93.3 % of the samples had mean DO ranging between 1.65 and 5.87 mg/l. As compared to the WHO acceptable standards for dissolved oxygen in fresh water (10–12 mg/l), the observed results were partly acceptable although significant number of individual records fall out of the range. Related observation was reported by Tenagne [44] from drinking water in Bahir Dar, Ethiopia, in which the mean DO concentration of the water samples were between 0.45 and 5.27 mg/l. Purushottam et al. [45], also reported DO values ranging from 1.2 to 4.6 mg/l from different lake water samples. Dissolved oxygen is an important water quality parameter and has special significance for aquatic organisms in natural waters [46]. Temperature of water influences the amount of dissolved oxygen with only lesser oxygen dissolved in warm water than cold water [44]. Therefore, high temperature of the water sources could be one of the factors for low DO values recorded in the current study.

The mean BOD after 5 days (BOD5) was found within the range of 8–77 mg/l. Although no guideline set for the maximum tolerable limit of BOD in drinking water, for fisheries and aquatic life, European Union and Ethiopia recommend 3–6 mg/l and less than 5 mg/l, respectively [43]. This suggests that drinking water sources were highly polluted by organic matter. Detection of phosphate in water sources (0.09–1.91 mg/l) usually indicates contamination of the water sources by run-off from agricultural farms using inorganic fertilizers [47]. Related result (0.27–1.41 mg/l) was also recorded from underground water samples from Ondo State, in the western part of Nigeria [48]. All the water samples assessed in this study were observed to have concentration of phosphate ions below the maximum permissible level (5 mg/l) set by European commission and WHO. The high phosphate concentrations in some of the water samples could be due to the presence of agricultural activities near the water sources, as most of the people in the study area were practicing farming. These observations indicate that the water from these sources could not be stored for long in open containers, as the presence of phosphate encourages the growth of algae and consequently cause adverse changes at least in colour and taste of the water sources [49].

The mean nitrate concentration in the samples varied from below detection limit to 102.11 mg/l. Accordingly, most of the water samples fall within the permissible limit (50 mg/l) set by the European commission [50] for drinkable water except for two of the protected wells with concentration above 50 mg/l. Study done on the quality of packaged water analyzed in Nigeria reported concentrations of 0.0–40.0 mg/l nitrate ions [51], while analysis on well water samples from the same country revealed nitrate concentration of about 50.6 mg/l [52]. Higher nitrate levels (>50 mg/l) were also previously reported [53]. These reports have conformity with the present findings. Similar observations have been reported from groundwater sources in Iganga, eastern Uganda, with nitrate levels ranged between 21 and 145 mg/l in protected springs. In another study done in Tanzania, nitrate levels ranging between 0 and 90.28 mg/l was recorded from different drinking water sources [54]. However, lower nitrate concentration was also reported from northeastern region of Buenos Aries Province, Argentina [55]. This variation may be explained by the differences in hydro-geological regimes and likely contaminant entry point. While nitrogen is a vital nutrient for plant growth, high concentrations are harmful to people and nature. The agricultural use of nitrates in organic and chemical fertilizers has been a major source of water pollution in Europe [50]. Generally, farming remains responsible for over 50 % of the total nitrogen discharge into surface waters. Thus, excessive nitrate concentrations in water are mainly related to pollution (with agriculture as the main source). Lifetime exposure to nitrite and nitrate at levels above the maximum acceptable concentration could cause such problems as diuresis, increased starch deposits and hemorrhaging of the spleen [53].

Because of their high toxicity to humans and aquatic life, some heavy metals have been used as indices of pollution [56]. The concentrations of metals ions, including lead, in the current water samples ranged from below detection level to 0.09 mg/l, with about 64.4 % of the water samples having lead concentration above the WHO maximum permissible level set for drinking waters [37]. Gebrekidan and Samuel [57] also reported Pb concentrations ranging from below detection level to 0.7 mg/l in ground drinking water in urban areas of Tigray, Ethiopia. Heavy metals have a marked effect on the aquatic flora and fauna which, through biomagnifications, enters the food chain and ultimately affect the human beings as well [58]. The heavy metals, in drinking water, are linked most often to human poisoning at larger dose are lead, iron, cadmium copper, zinc, chromium etc. The known fatal effects of heavy metal toxicity in drinking water include damaged or reduced mental and central nervous function and lower energy level.

Similar to the case of lead, zinc concentration ranging between below detection level to maximum of 0.27 mg/l were recorded from the different water samples. As compared to the maximum permissible level of the same in surface water (0.01 mg/l) and ground water (0.05 mg/l) [37], the observed zinc concentrations were significantly high with the concentration being much higher due to dissolution of zinc from the used pipes. However, the overall result recorded in this study showed that all the samples had Zn concentration within Ethiopian maximum permissible level (5 mg/l).

## Conclusion

Bacteriological quality of most water samples analyzed in the current study did not meet the standards set for drinking water. From the quality and sanitary risk evaluation points of view, the studied water sources could be classified as grossly polluted and only very few of them had reasonable quality. Most of the physico-chemical data indicated marginally tolerable quality with respect to pH and TSS but poor quality in relation to turbidity, temperature, conductivity, BOD and nitrate concentration with values much in excess of the permissible standards. Excessive nitrate concentrations recoded from some water samples are mainly related to pollution (with agriculture as the main source). Lifetime exposure to nitrite and nitrate at levels above the maximum acceptable concentration could cause many health problems including increased starch deposits and hemorrhaging of the spleen. Lead concentrations recorded in most of water sources were above the permissible level stated in many guide lines. Thus, with the current high dependence on alternative water sources other than tap water, it calls for awareness development on hygienic handling of wells and springs besides designing protections and regular purification strategies by the concerned bodies.

## Additional files

10.1186/s13104-015-1376-5. The status and care being given to drinking water sources (n = 237) in Serbo town and its surroundings, 2012.
10.1186/s13104-015-1376-5. The prevalence of *Salmonella* and *Shigella* in drinking water samples, Serbo town and its surroundings, 2012 (n = 3 for each sample sites).
10.1186/s13104-015-1376-5. Correlations among measured parameters of water samples from five different water sources, Serbo town and its surroundings, 2012.
