# Supplementary material for: Physico-chemical and bacteriological quality of drinking water of different sources, Jimma zone, Southwest Ethiopia
Source: BMC Res Notes. 2015 Oct 5;8:541. doi: 10.1186/s13104-015-1376-5 (PMC4594903; doi:10.1186/s13104-015-1376-5)
Supplement: Supplementary file 1 — 10.1186/s13104-015-1376-5. The status and care being given to drinking water sources (n = 237) in Serbo town and its surroundings, 2012. [file 13104_2015_1376_MOESM1_ESM.doc]

**Table S1. The status and care being given to drinking water sources (n=237) in Serbo town and its surroundings, 2012**

| **Characteristics** | | **No of respondents** | **%** |
| --- | --- | --- | --- |
| Source of drinking water | Unprotected spring | 76 | 32.1 |
|  | Unprotected well | 44 | 18.6 |
|  | Tap water | 43 | 18.1 |
|  | Protected well | 37 | 15.6 |
|  | Protected spring | 40 | 16.9 |
| Type of container used to collect water | Bucket | 26 | 11.0 |
|  | Plastic pot | 205 | 86.5 |
|  | Clay pot | 6 | 2.5 |
| Method of purification of water before use | Directly used | 231 | 97.5 |
|  | Boiling | 6 | 2.5 |
| Distance of water source from latrine (m) | 5-10 | 16 | 6.7 |
|  | 11-15 | 35 | 14.8 |
|  | 16-20 | 84 | 35.4 |
|  | >20 | 102 | 43.0 |
| Position of water in relation to latrine | Above | 26 | 11.0 |
|  | Below | 76 | 32.1 |
|  | Parallel | 128 | 54.0 |
| Waste disposal method | Bury in pit | 50 | 21.1 |
|  | Open disposal | 134 | 56.5 |
|  | Burning | 53 | 22.4 |
| Common diseases in the study area | Diarrhea | 95 | 40.1 |
|  | Malaria | 107 | 45.1 |
|  | Others | 35 | 14.8 |
