# Supplementary material for: Physico-chemical and bacteriological quality of drinking water of different sources, Jimma zone, Southwest Ethiopia
Source: BMC Res Notes. 2015 Oct 5;8:541. doi: 10.1186/s13104-015-1376-5 (PMC4594903; doi:10.1186/s13104-015-1376-5)
Supplement: Supplementary file 2 — 10.1186/s13104-015-1376-5. The prevalence of Salmonella and Shigella in drinking water samples, Serbo town and its surroundings, 2012 (n = 3 for each sample sites). [file 13104_2015_1376_MOESM2_ESM.doc]

Table S2: The prevalence of *Salmonella* and *Shigella* in drinking water samples, Serbo town and its surroundings, 2012 (n = 3 for each sample sites).

| **Type of water source** | **Sample size** | ***Salmonella* positive** | | ***Shigella* positive** | |
| --- | --- | --- | --- | --- | --- |
|  |  | No | % | No | % |
| Tap water | 15 | 0 | 0 | 0 | 0 |
| Protected wells | 15 | 0 | 0 | 0 | 0 |
| Unprotected wells | 18 | 1 | 5.60 | 0 | 0 |
| Protected spring | 15 | 0 | 0 | 0 | 0 |
| Unprotected spring | 27 | 2 | 7.41 | 0 | 0 |
| Total | 90 | 3 | 3.33 | 0 | 0 |
