# Supplementary material for: Physico-chemical and bacteriological quality of drinking water of different sources, Jimma zone, Southwest Ethiopia
Source: BMC Res Notes. 2015 Oct 5;8:541. doi: 10.1186/s13104-015-1376-5 (PMC4594903; doi:10.1186/s13104-015-1376-5)
Supplement: Supplementary file 3 — 10.1186/s13104-015-1376-5. Correlations among measured parameters of water samples from five different water sources, Serbo town and its surroundings, 2012. [file 13104_2015_1376_MOESM3_ESM.doc]

Table S3: Correlations among measured parameters of water samples from five different water sources, Serbo town and its surroundings, 2012.

|  |  | AMC | Temp | pH | EC | Turbidity | DO | PO4^-^ | NO3- | TSS | TDS |
| --- | --- | --- | --- | --- | --- | --- | --- | --- | --- | --- | --- |
| AMC | R | 1 | -0.624 | -0.829^*^ | -0.845^*^ | 0.721 | 0.626 | -0.244 | -0.315 | 0.718 | -0.972^**^ |
|  | P-value |  | 0.13 | 0.041 | 0.036 | 0.084 | 0.129 | 0.346 | 0.303 | 0.086 | 0.003 |
| Temp | R | -0.624 | 1 | 0.557 | 0.673 | -0.987^**^ | 0.064 | 0.178 | 0.407 | -0.243 | 0.445 |
|  | P-value | 0.13 |  | 0.165 | 0.107 | 0.001 | 0.459 | 0.387 | 0.248 | 0.347 | 0.226 |
| pH | R | -0.829^*^ | 0.557 | 1 | .838^*^ | -0.647 | -0.563 | 0.558 | 0 | -0.607 | 0.851^*^ |
|  | P-value | 0.041 | 0.165 |  | 0.038 | 0.119 | 0.161 | 0.164 | 0.5 | 0.139 | 0.034 |
| EC | R | -0.845^*^ | 0.673 | 0.838^*^ | 1 | -0.705 | -0.675 | 0.032 | 0.544 | -0.281 | 0.831^*^ |
|  | P-value | 0.036 | 0.107 | 0.038 |  | 0.092 | 0.106 | 0.479 | 0.172 | 0.324 | 0.041 |
| Turbidity | R | 0.721 | -.987^**^ | -0.647 | -0.705 | 1 | 0.012 | -0.277 | -0.335 | 0.395 | -0.558 |
|  | P-value | 0.084 | 0.001 | 0.119 | 0.092 |  | 0.492 | 0.326 | 0.291 | 0.255 | 0.164 |
| DO | R | 0.626 | 0.064 | -0.563 | -0.675 | 0.012 | 1 | 0.148 | -0.358 | 0.284 | -0.77 |
|  | P-value | 0.129 | 0.459 | 0.161 | 0.106 | 0.492 |  | 0.406 | 0.277 | 0.322 | 0.064 |
| PO^3-^4 | R | -0.244 | 0.178 | 0.558 | 0.032 | -0.277 | 0.148 | 1 | -0.787 | -0.667 | 0.252 |
|  | P-value | 0.346 | 0.387 | 0.164 | 0.479 | 0.326 | 0.406 |  | 0.057 | 0.11 | 0.341 |
| NO3- | R | -0.315 | 0.407 | 0 | 0.544 | -0.335 | -0.358 | -0.787 | 1 | 0.359 | 0.245 |
|  | P-value | 0.303 | 0.248 | 0.5 | 0.172 | 0.291 | 0.277 | 0.057 |  | 0.276 | 0.345 |
| TSS | R | 0.718 | -0.243 | -0.607 | -0.281 | 0.395 | 0.284 | -0.667 | 0.359 | 1 | -0.719 |
|  | P-value | 0.086 | 0.347 | 0.139 | 0.324 | 0.255 | 0.322 | 0.11 | 0.276 |  | 0.086 |
| TDS | R | -0.972^**^ | 0.445 | 0.851^*^ | 0.831^*^ | -0.558 | -0.77 | 0.252 | 0.245 | -0.719 | 1 |
|  | P-value | 0.003 | 0.226 | 0.034 | 0.041 | 0.164 | 0.064 | 0.341 | 0.345 | 0.086 |  |
| BOD | R | 0.542 | -0.813^*^ | -0.557 | -0.351 | 0.860^*^ | -0.279 | -0.653 | 0.165 | 0.6 | -0.383 |
|  | P-value | 0.173 | 0.047 | 0.165 | 0.281 | 0.031 | 0.325 | 0.116 | 0.395 | 0.142 | 0.262 |

*****. Correlation is significant at 0.05 level (1-tailed). ******. Correlation is significant at 0.01 level (1-tailed).
